# Comparative transcriptional profiling of renal cortex in rats with inherited stress-induced arterial hypertension and normotensive Wistar Albino Glaxo rats

**DOI:** 10.1186/s12863-015-0306-9

**Published:** 2016-01-27

**Authors:** Larisa A. Fedoseeva, Marina A. Ryazanova, Nikita I. Ershov, Arcady L. Markel, Olga E. Redina

**Affiliations:** Institute of Cytology and Genetics, Siberian Branch of Russian Academy of Sciences, Novosibirsk, Russian Federation; Novosibirsk State University, Novosibirsk, Russian Federation

**Keywords:** Stress-sensitive hypertension, Renal cortex, Transcriptional profiling, RNA-Seq, ISIAH rats

## Abstract

**Background:**

The renal function plays a leading role in long-term control of arterial pressure. The comparative analysis of renal cortex transcriptome in ISIAH rats with inherited stress-induced arterial hypertension and normotensive WAG rats was performed using RNA-Seq approach. The goal of the study was to identify the differentially expressed genes (DEGs) related to hypertension and to detect the pathways contributing to the differences in renal functions in ISIAH and WAG rats.

**Results:**

The analysis revealed 716 genes differentially expressed in renal cortex of ISIAH and WAG rats, 42 of them were associated with arterial hypertension and regulation of blood pressure (BP). Several Gene Ontology (GO) terms significantly enriched with DEGs suggested the existence of the hormone dependent interstrain differences in renal cortex function. Multiple DEGs were associated with regulation of blood pressure and blood circulation, with the response to stress (including oxidative stress, hypoxia, and fluid shear stress) and its regulation. Several other processes which may contribute to hypertension development in ISIAH rats were: ion transport, regulation of calcium ion transport, homeostatic process, tissue remodeling, immune system process and regulation of immune response.

KEGG analysis marked out several pathways significantly enriched with DEGs related to immune system function, to steroid hormone biosynthesis, tryptophan, glutathione, nitrogen, and drug metabolism.

**Conclusions:**

The results of the study provide a basis for identification of potential biomarkers of stress-sensitive hypertension and for further investigation of the mechanisms that affect renal cortex function and hypertension development.

**Electronic supplementary material:**

The online version of this article (doi:10.1186/s12863-015-0306-9) contains supplementary material, which is available to authorized users.

## Background

The etiology of essential hypertension is multifactorial. Many researchers agree that the renal function plays a leading role in long-term control of arterial pressure. It is thought that hypertension may be a consequence of abnormal water-electrolyte balancing by kidney [1, 2]. However, the number of factors involved in the pathogenesis of hypertension is constantly increasing [3], and the molecular mechanisms of essential hypertension development still remain uncertain.

The use of experimental animal models provides valuable information to elucidate the nature of polygenic traits [4]. One of these is the ISIAH (Inherited Stress-Induced Arterial Hypertension) rat strain which was developed to study the mechanisms of the stress-sensitive hypertension and its complications. The ISIAH rats were selected from an outbred Wistar rat population for a systolic arterial blood pressure (SABP) increase induced by 30 min restraint stress in a small cylindrical wire mesh cage. More than 30 generations of close inbreeding by brother-sister mating resulted in a high genetic homogeneity of the ISIAH strain [5]. Starting from the age of 6 weeks, the ISIAH rats have elevated SABP at basal condition (175.0 ± 3.5 mmHg in males and 165.0 ± 3.0 mmHg in females) and exhibit a dramatic increase in SABP (up to 210 mmHg and more) when restrained [6, 7]. The ISIAH rats also show a number of other characteristic features of hypertensive state: hypertrophy of the left ventricle, increase in the wall thickness of the small arteries, and changes in the electrocardiographic pattern [7]. ISIAH rats are also characterized by increased kidney mass [8] and some alterations in kidney histology indicative of increase in filtration barrier functional load and of initial stages of glomerular [9] and renomedullar sclerosis [10]. These features, the genetically determined enhanced responsiveness to stressful stimulation, and the predominant involvement of the neuroendocrine hypothalamic-pituitary-adrenocortical (HPA) and sympathoadrenal systems during the disease development let to consider the ISIAH rat strain as an advantageous model of the human stress-sensitive hypertensive state [11].

Recently, the next-generation sequencing technologies became very useful in providing deep insights into molecular mechanisms underlying the complex diseases development [12]. In the current work, the RNA sequencing (RNA-Seq) technology was used for comparative analysis of renal cortex transcriptome in hypertensive ISIAH and normotensive control WAG (Wistar Albino Glaxo) rats. The goal of the study was to identify the differentially expressed genes (DEGs) related to hypertension and to detect the pathways contributing to the differences in renal functions in hypertensive ISIAH and normotensive WAG rats.

The study revealed multiple DEGs in renal cortex of hypertensive ISIAH and normotensive WAG rats, including 42 DEGs known as related to hypertension and regulation of BP. These DEGs were associated with the diversity of biological processes and pathways which might contribute to development of stress-sensitive hypertension. Two of them (*Ephx2 and Glp1r*) were in the list of the top 40 genes showing the highest differences in expression in ISIAH and WAG renal cortex.

## Results

The analysis revealed 716 genes differentially expressed in the renal cortex of hypertensive ISIAH and normotensive WAG rats. About the half of DEGs (372 genes, i.e. 52,0 %) were downregulated in ISIAH renal cortex. The expression of nine of these genes was detected in renal cortex of WAG rats but not in ISIAH. Alternatively, only one gene was expressed in ISIAH and silent in WAG renal cortex (Table 1). No one of these genes expressed in renal cortex of only one rat strain is known as related to hypertension development. The list of the top 40 genes showing the highest differences in expression in ISIAH and WAG renal cortex contained two genes (*Ephx2 and Glp1r*) associated with hypertension (Table 2).

**Table 1:** Genes expressed at detectable levels in renal cortex of only one of rat strains

Gene symbol	Acc.#	Value, FPKM		Gene name	q-value
		WAG	ISIAH		
*Cyp2c24*	NM_001271354.1	10.30	0	Cytochrome P450, family 2, subfamily c, polypeptide 24	0.002
*Hpse2*	NM_001135762.1	1.34	0	Heparanase 2	0.002
*LOC100362965*	XM_002728491.2	5.57	0	SNRPN upstream reading frame protein-like	0.002
*LOC102546948*	XR_352663.1	7.28	0	Uncharacterized LOC102546948, transcript variant X2	0.002
*LOC102547398*	XR_358422.1	9.97	0	Uncharacterized LOC102547398	0.002
*LOC102553584*	XR_362425.1	1.87	0	Uncharacterized LOC102553584	0.002
*RGD1309362*	NM_001024884.1	2.45	0	Similar to interferon-inducible GTPase	0.002
*Sfta2*	NM_001166020.1	8.22	0	Surfactant associated 2	0.003
*Slpil2*	NM_001008872.1	2.25	0	Antileukoproteinase-like 2	0.029
*Rpl38-ps1*	XR_593941.1	0	2.45	Ribosomal protein L38, pseudogene 1	0.030

**Table 2:** Top 40 genes with the greatest difference in expression between ISIAH and WAG renal cortices

Gene symbol	Acc. #	Gene name	log2 fold change ISIAH/WAG
*RGD1565131*	XM_006248902.1	60S ribosomal protein L15-like	8.57
*Fam111a*	NM_001109163.1	Family with sequence similarity 111, member A	5.35
*Stk32c*	XM_006230485.1	Serine/threonine kinase 32C, transcript variant X1	4.91
*Resp18*	NM_019278.1	Regulated endocrine-specific protein 18	4.82
*Ubd*	NM_053299.2	Ubiquitin D	4.55
*Ephx2* ^a^	XM_006252147.1	Epoxide hydrolase 2, cytoplasmic, transcript variant X1	4.48
*Akr1b8*	XM_006236251.1	Aldo-keto reductase family 1, member B8, transcript variant X1	4.45
*Hpgd*	NM_024390.2	Hydroxyprostaglandin dehydrogenase 15 (NAD)	4.30
*Ly6al*	XM_006241767.1	Lymphocyte antigen 6 complex, locus A-like, transcript variant X1	4.14
*Spta1*	NM_001011908.3	Spectrin, alpha, erythrocytic 1 (elliptocytosis 2)	4.07
*Tcerg1l*	NM_001130077.1	Transcription elongation regulator 1-like	3.91
*Glp1r* ^a^	XR_362044.1	Glucagon-like peptide 1 receptor, transcript variant X1	3.79
*LOC686967*	XM_003749071.2	Similar to olfactory receptor 1442	3.72
*LOC102551856*	XR_353697.1	Uncharacterized LOC102551856, transcript variant X1	3.46
*Mx2*	XM_006248150.1	Myxovirus (influenza virus) resistance 2, transcript variant X1	3.40
*Krt19*	NM_199498.1	Keratin 19	3.37
*Ppp2r2c*	NM_057116.1	Protein phosphatase 2, regulatory subunit B, gamma	3.27
*G6b*	XM_006256069.1	G6b protein, transcript variant X1	3.09
*LOC102555352*	XR_350674.1	Uncharacterized LOC102555352, transcript variant X4	3.05
*RGD1564278*	XM_003749906.2	RNA-binding protein with serine-rich domain 1-like	3.02
*Kcne1*	XM_006248034.1	Potassium voltage-gated channel, Isk-related family, member 1, transcript variant X2	2.89
*LOC102546968*	XM_006256098.1	RT1 class I histocompatibility antigen, AA alpha chain-like	2.87
*Grhl1*	XM_234006.7	Grainyhead-like 1 (Drosophila)	2.85
*Fat3*	XM_006242552.1	FAT atypical cadherin 3, transcript variant X1	−2.86
*Fabp4*	NM_053365.1	Fatty acid binding protein 4, adipocyte	−3.23
*Rbp4*	NM_013162.1	Retinol binding protein 4, plasma	−3.29
*LOC361914*	NM_001017465.1	Similar to solute carrier family 7 (cationic amino acid transporter, y + system), member 12	−3.32
*Cyp24a1*	XM_006235672.1	Cytochrome P450, family 24, subfamily a, polypeptide 1, transcript variant X1	−3.47
*B3gat1*	XM_006242733.1	Beta-1,3-glucuronyltransferase 1 (glucuronosyltransferase P), transcript variant X1	−3.47
*LOC102552001*	XR_350962.1	Uncharacterized LOC102552001, transcript variant X1	−3.60
*Hhip*	NM_001191817.1	Hedgehog-interacting protein	−3.64
*Slc22a13*	XM_006244092.1	Solute carrier family 22 (organic anion/urate transporter), member 13, transcript variant X1	−3.69
*LOC102546318*	XR_361882.1	Uncharacterized LOC102546318	−3.97
*LOC102548532*	XR_360708.1	Uncharacterized LOC102548532	−4.00
*LOC102553060*	XR_362149.1	Uncharacterized LOC102553060, transcript variant X1	−4.10
*LOC102550987*	XR_360671.1	Uncharacterized LOC102550987	−4.11
*LOC501110*	NM_001024361.1	Similar to Glutathione S-transferase A1 (GTH1) (HA subunit 1) (GST-epsilon) (GSTA1-1) (GST class-alpha)	−4.26
*Pcdh9*	NM_001191688.1	Protocadherin 9	−4.67
*Pdilt*	NM_001013902.1	Protein disulfide isomerase-like, testis expressed	−5.06
*LOC100360791*	XM_003748668.2	Tumor protein, translationally-controlled 1	−7.45

^a^-genes associated with hypertension

Altogether, the study revealed 39 DEGs annotated in RGD as related to hypertension (Table 3). Six of these genes (*Ace, Cyp2j4, Gja1, Mmp9, Ppara*, and *Ren*) were described as genes associated with renal hypertension. According to functional annotation in DAVID, three additional DEGs (*Guca2b, P2rx4, and Pcsk5)* might be associated with regulation of BP. These DEGs may be considered as potential candidate genes related to blood pressure complications in ISIAH rats. Most of these genes were downregulated in hypertensive kidney. The majority of the DEGs associated with hypertension were related to insulin resistance and diabetic nephropathy and about half of them were associated with the immune system diseases (Table 3). Altogether, the study revealed 60 DEGs referred in RGD as associated with renal diseases, including renal fibrosis, renal insufficiency, glomerulonephritis, diabetic nephropathy, and nephrosclerosis (Table 4).

**Table 3:** Genes differentially expressed in ISIAH versus WAG renal cortex and annotated in databases as associated with hypertension and blood pressure regulation

Gene symbol	Acc. #	Gene name	log2 fold change ISIAH/WAG
Rat genome database			
*Ace* ^a^ ^bc^	NM_012544.1	Angiotensin I converting enzyme	−1.18
*Acsm3* ^c^	XM_006230106.1	Acyl-CoA synthetase medium-chain family member 3, transcript variant X2	2.51
*Adra1b* ^b^	NM_016991.2	Adrenoceptor alpha 1B	−1.24
*Adra2a*	NM_012739.3	Adrenoceptor alpha 2A	−1.01
*Alas1*	NM_024484.2	Aminolevulinate, delta-, synthase 1	0.47
*Angpt1*	XM_006241609.1	Angiopoietin 1, transcript variant X1	0.69
*Apob* ^a^ ^bc^	NM_019287.2	Apolipoprotein B	−2.52
*Arg2* ^a^ ^c^	NM_019168.1	Arginase 2	−0.58
*Cdo1*	XM_006254698.1	Cysteine dioxygenase type 1, transcript variant X1	−0.78
*Clu* ^a^ ^c^	XM_006252094.1	Clusterin, transcript variant X1	−1.75
*Comt*	NM_012531.2	Catechol-O-methyltransferase	−0.82
*Cst3* ^a^ ^bc^	NM_012837.1	Cystatin C	−0.50
*Cyp1a1* ^c^	NM_012540.2	Cytochrome P450, family 1, subfamily a, polypeptide 1	−1.04
*Cyp2j4* ^a^	NM_023025.2	Cytochrome P450, family 2, subfamily j, polypeptide 4	−0.81
*Cyp4a8*	NM_031605.2	Cytochrome P450, family 4, subfamily a, polypeptide 8	−0.55
*Ephx1* ^c^	NM_012844.3	Epoxide hydrolase 1, microsomal (xenobiotic), transcript variant 2	0.59
*Ephx2* ^a^ ^b^	XM_006252147.1	Epoxide hydrolase 2, cytoplasmic, transcript variant X1	4.48
*Gja1* ^c^	XM_006256503.1	Gap junction protein, alpha 1, transcript variant X2	0.54
*Glp1r*	XR_362044.1	Glucagon-like peptide 1 receptor, transcript variant X1	3.79
*Hsd11b2* ^c^	NM_017081.2	Hydroxysteroid 11-beta dehydrogenase 2	0.60
*Itgav* ^b^	XM_006234437.1	Integrin, alpha V	−0.62
*Klk1c12* ^ab^	NM_001005382.1	Kallikrein 1-related peptidase C12	−1.86
*Klkb1* ^ac^	XM_006253121.1	Kallikrein B, plasma 1, transcript variant X1	2.22
*Mif* ^bc^	NM_031051.1	Macrophage migration inhibitory factor (glycosylation-inhibiting factor)	0.50
*Mmp9* ^ac^	XM_006235619.1	Matrix metallopeptidase 9, transcript variant X1	−2.49
*Mthfr* ^ac^	XM_006239414.1	Methylenetetrahydrofolate reductase (NAD(P)H), transcript variant X2	0.49
*Ppara* ^bc^	XM_006242154.1	Peroxisome proliferator activated receptor alpha, transcript variant X4	−0.56
*Ptgds* ^a^ ^b^	NM_013015.2	Prostaglandin D2 synthase (brain)	−0.67
*Ptk2b* ^bc^	XM_006252145.1	Protein tyrosine kinase 2 beta, transcript variant X3	−0.51
*Ren* ^a^	NM_012642.4	Renin	−0.49
*RT1-Bb* ^c^	NM_001004084.2	RT1 class II, locus Bb	−0.78
*Slc26a4*	XM_006239978.1	Solute carrier family 26 (anion exchanger), member 4, transcript variant X3	0.46
*Slc2a4* ^b^	NM_012751.1	Solute carrier family 2 (facilitated glucose transporter), member 4	−0.77
*Slc9a3r2*	XM_006245893.1	Solute carrier family 9, subfamily A (NHE3, cation proton antiporter 3), member 3 regulator 2, transcript variant X2	−0.48
*Sncg*	NM_031688.1	Synuclein, gamma (breast cancer-specific protein 1)	−1.25
*Sts*	XM_006256960.1	Steroid sulfatase (microsomal), isozyme S, transcript variant X1	−0.70
*Tf* ^a^ ^bc^	NM_001013110.1	Transferrin	−1.50
*Vwf* ^bc^	NM_053889.1	Von Willebrand factor	0.77
*Xdh* ^a^ ^c^	NM_017154.1	Santhine dehydrogenase	−0.77
David (regulation of blood pressure)			
*Guca2b*	NM_022284.2	Guanylate cyclase activator 2B	2.04
*P2rx4*	NM_031594.1	Purinergic receptor P2X, ligand-gated ion channel 4	−1.65
*Pcsk5*	XM_006231145.1	Proprotein convertase subtilisin/kexin type 5, transcript variant X2	0.53

Genes associated with: ^a^-diabetic nephropathy; ^b^-insulin resistance; ^c^ *-*immune system diseases

**Table 4:** Genes differentially expressed in ISIAH versus WAG renal cortex and annotated in rat genome database as associated with kidney diseases

Gene symbol	Acc. #	Gene name	log2 fold change ISIAH/WAG
*Abcb1a* ^b^	NM_133401.1	ATP-binding cassette, sub-family B (MDR/TAP), member 1A	−0.82
*Abcc2* ^b^	NM_012833.1	ATP-binding cassette, subfamily C (CFTR/MRP), member 2	−0.47
*Ace* ^abcd^	NM_012544.1	Angiotensin I converting enzyme	−1.18
*Acsm3* ^c^	XM_006230106.1	Acyl-CoA synthetase medium-chain family member 3, transcript variant X2	2.51
*Aif1* ^c^	NM_017196.3	Allograft inflammatory factor 1	0.59
*Angpt1*	XM_006241609.1	Angiopoietin 1, transcript variant X1	0.69
*Apob* ^d^	NM_019287.2	Apolipoprotein B	−2.52
*Apoc2* ^b^	XM_006228403.1	Apolipoprotein C-II, transcript variant X1	−1.20
*Apoh* ^d^	NM_001009626.1	Apolipoprotein H (beta-2-glycoprotein I)	−2.73
*Arg2* ^bd^	NM_019168.1	Arginase 2	−0.58
*Atp6v1b1*	XM_006236748.1	ATPase, H transporting, lysosomal V1 subunit B1, transcript variant X1	0.57
*Bak1*	NM_053812.1	BCL2-antagonist/killer 1	0.54
*Bsnd*	NM_138979.2	Bartter syndrome, infantile, with sensorineural deafness (Barttin)	0.45
*C1qa* ^c^	NM_001008515.1	Complement component 1, q subcomponent, A chain	0.59
*Cfb* ^abc^	NM_212466.3	Complement factor B	−1.09
*Clu* ^abcd^	XM_006252094.1	Clusterin, transcript variant X1	−1.75
*Cndp1* ^cd^	NM_001007687.1	Carnosine dipeptidase 1 (metallopeptidase M20 family)	−1.01
*Col3a1* ^ab^	NM_032085.1	Collagen, type III, alpha 1	−0.60
*Comt*	NM_012531.2	Catechol-O-methyltransferase	−0.82
*Cst3* ^d^	NM_012837.1	Cystatin C	−0.50
*Cyp1a1* ^b^	NM_012540.2	Cytochrome P450, family 1, subfamily a, polypeptide 1	−1.04
*Cyp2j4* ^ad^	NM_023025.2	Cytochrome P450, family 2, subfamily j, polypeptide 4	−0.81
*Cyp4a8*	NM_031605.2	Cytochrome P450, family 4, subfamily a, polypeptide 8	−0.55
*Ephx2* ^bd^	XM_006252147.1	Epoxide hydrolase 2, cytoplasmic, transcript variant X1	4.48
*Fga* ^b^	NM_001008724.1	Fibrinogen alpha chain, transcript variant 1	1.00
*Fhit*	NM_021774.1	Fragile histidine triad	0.80
*Fmod* ^d^	XM_006249885.1	Fibromodulin, transcript variant X1	−0.94
*Gatm* ^b^	NM_031031.2	Glycine amidinotransferase (L-arginine:glycine amidinotransferase)	0.74
*Gfpt2* ^d^	NM_001002819.2	Glutamine-fructose-6-phosphate transaminase 2	1.75
*Gja1*	XM_006256503.1	Gap junction protein, alpha 1, transcript variant X2	0.54
*Gtpbp4* ^b^	XM_006254146.1	GTP binding protein 4	1.77
*Hao1*	XM_006235096.1	Hydroxyacid oxidase (glycolate oxidase) 1, transcript variant X1	−1.68
*Igfbp1* ^d^	NM_013144.1	Insulin-like growth factor binding protein 1	1.09
*Itgal*	XM_006230269.1	Integrin, alpha L, transcript variant X1	0.71
*Kit*	XM_006250909.1	v-kit Hardy-Zuckerman 4 feline sarcoma viral oncogene homolog, transcript variant X1	0.62
*Klk1c12* ^d^	NM_001005382.1	Kallikrein 1-related peptidase C12	−1.86
*Klkb1* ^cd^	XM_006253121.1	Kallikrein B, plasma 1, transcript variant X1	2.22
*Lgals1*	NM_019904.1	Lectin, galactoside-binding, soluble, 1	0.91
*Mif* ^c^	NM_031051.1	Macrophage migration inhibitory factor (glycosylation-inhibiting factor)	0.50
*Mme* ^c^	NM_012608.2	Membrane metallo-endopeptidase	0.55
*Mmp9* ^acd^	XM_006235619.1	Matrix metallopeptidase 9, transcript variant X1	−2.49
*Mthfr* ^bcde^	XM_006239414.1	Methylenetetrahydrofolate reductase (NAD(P)H), transcript variant X2	0.49
*Muc20* ^bc^	XM_006248449.1	Mucin 20, cell surface associated, transcript variant X1	0.63
*Pla2g7* ^abc^	XM_006244606.1	Phospholipase A2, group VII (platelet-activating factor acetylhydrolase, plasma), transcript variant X1	0.71
*Ppara* ^c^	XM_006242154.1	Peroxisome proliferator activated receptor alpha, transcript variant X4	−0.56
*Ptgds* ^de^	NM_013015.2	Prostaglandin D2 synthase (brain)	−0.67
*Ptk2b* ^c^	XM_006252145.1	Protein tyrosine kinase 2 beta, transcript variant X3	−0.51
*Ren* ^bd^	NM_012642.4	Renin	−0.49
*Rhcg* ^b^	NM_183053.1	Rh family, C glycoprotein	0.66
*RT1-Bb* ^a^	NM_001004084.2	RT1 class II, locus Bb	−0.78
*Serpinf1* ^d^	NM_177927.2	Serpin peptidase inhibitor, clade F (alpha-2 antiplasmin, pigment epithelium derived factor), member 1	−1.34
*Slc17a2*	NM_001107353.1	Solute carrier family 17, member 2	0.52
*Slc19a3* ^b^	NM_001108228.1	Solute carrier family 19 (thiamine transporter), member 3	0.66
*Slc4a1*	XM_006247255.1	Solute carrier family 4 (anion exchanger), member 1, transcript variant X1	0.86
*Tf* ^cd^	NM_001013110.1	Transferrin	−1.50
*Tgfbi* ^d^	NM_053802.1	Transforming growth factor, beta induced	−0.93
*Ttc21b*	NM_001191737.1	Tetratricopeptide repeat domain 21B	−0.48
*Vwf* ^abc^	NM_053889.1	Von Willebrand factor	0.77
*Wfs1*	NM_031823.1	Wolfram syndrome 1 (wolframin)	0.50
*Xdh* ^abd^	NM_017154.1	Xanthine dehydrogenase	−0.77

Genes associated with: ^a^-renal fibrosis; ^b^ *-*renal insufficiency; ^c^-glomerulonephritis, ^d^-diabetic nephropathies; ^e^-nephrosclerosis

Thirty one transcription factor genes were differentially expressed in ISIAH and WAG renal cortex (Table 5). One of these (*Ppara*) is known as associated with hypertension, glomerulonephritis, insulin resistance, and immune system diseases. Its expression was downregulated in ISIAH rats.

**Table 5:** List of genes encoding transcription factors differentially expressed in ISIAH versus WAG renal cortex

Gene symbol	Acc. #	Gene name	log2 fold change ISIAH/WAG
*Bcl6*	NM_001107084.1	B-cell CLL/lymphoma 6	−2.47
*Btbd11*	XM_006241173.1	BTB (POZ) domain containing 11	−0.98
*Etv1*	XM_006240048.1	Ets variant 1, transcript variant X2	−0.85
*Etv5*	XM_006248542.1	Ets variant 5, transcript variant X1	−0.84
*Foxi1*	NM_001105776.1	Forkhead box I1	0.48
*Grhl1*	XM_234006.7	Grainyhead-like 1 (Drosophila)	2.85
*Hdac9*	XM_006240037.1	Histone deacetylase 9, transcript variant X14	−0.89
*Hes6*	XM_006245407.1	Hes family bHLH transcription factor 6, transcript variant X1	−0.72
*Hr*	XM_006252284.1	Hair growth associated, transcript variant X2	1.24
*Irf4*	XM_006253900.1	Interferon regulatory factor 4, transcript variant X2	−1.51
*Irf7*	NM_001033691.1	Interferon regulatory factor 7	1.52
*Ivns1abp*	XM_006249989.1	Influenza virus NS1A binding protein, transcript variant X2	−0.59
*Klf12*	NM_001007684.1	Kruppel-like factor 2	−0.69
*Mybl1*	XM_006237749.1	Myeloblastosis oncogene-like 1, transcript variant X1	−1.37
*Nfkbil1*	XM_006256046.1	Nuclear factor of kappa light polypeptide gene enhancer in B-cells inhibitor-like 1, transcript variant X1	0.94
*Nkd2*	NM_001107454.1	Naked cuticle homolog 2 (Drosophila)	−0.84
*Osr2*	XM_006241542.1	Odd-skipped related transciption factor 2, transcript variant X1	1.03
*P8*	XM_006230201.1	Nuclear proten 1	−1.86
*Pou2af1*	NM_001109599.1	POU class 2 associating factor 1	2.60
*Ppara*	XM_006242154.1	Peroxisome proliferator activated receptor alpha, transcript variant X4	−0.56
*Ppargc1b*	NM_176075.2	Peroxisome proliferator-activated receptor gamma, coactivator 1 beta	−0.62
*Prox1*	XM_006250454.1	Prospero homeobox 1, transcript variant X1	−0.74
*Satb2*	XM_006244931.1	SATB homeobox 2, transcript variant X1	−1.13
*Sox9*	XM_003750950.2	SRY (sex determining region Y)-box 9	−0.80
*Spry4*	XM_006254656.1	Sprouty homolog 4 (Drosophila), transcript variant X2	−0.70
*Tcerg1l*	NM_001130077.1	Transcription elongation regulator 1-like	3.91
*Tcf4*	NM_053369.1	Transcription factor 4	−0.90
*Zbtb16*	XM_006243015.1	Zinc finger and BTB domain containing 16, transcript variant X1	−1.20
*Zdhhc2*	NM_145096.2	Zinc finger, DHHC-type containing 2	0.67
*Zfp36*	NM_133290.3	Zinc finger protein 36	−0.61
*Zfp385b*	NM_001107736.1	Zinc finger protein 385B	0.67

**Fig. 1:**
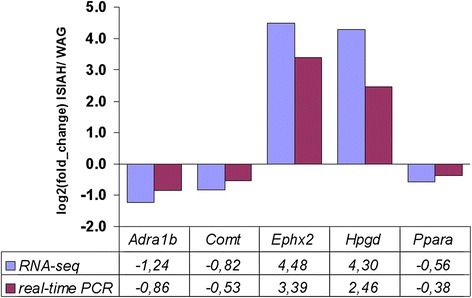
Comparison of gene expression level measurements obtained by RNA-seq and qPCR

Several genes which might play a key role in hypertension development in ISIAH rats were chosen for technical validation of the difference in their transcriptional activity in ISIAH and WAG renal cortex by real-time PCR (Fig. 1). The correlation coefficient between the results of two methods was 0.99.

Gene Ontology (GO) terms for biological processes found to be significantly enriched are represented in Additional file 1. As it is marked in this file, the majority of gene groups defined by GO terms contain DEGs associated with hypertension. Several groups of DEGs which might be important for the development of the stress-sensitive hypertension are given in bold in the file. The genes in these groups are listed in Additional file 2. Among the most significantly enriched GO terms, there are several (regulation of hormone levels, hormone metabolic processes, response to hormone and to insulin stimuli) which suggest the existence of the hormone dependent interstrain differences in renal cortex function.

Multiple DEGs were related to the response to stress and regulation of response to stress. The particular types of stress were specified by several GO terms such as response to oxidative stress, response to hypoxia, and response to fluid shear stress. In these groups, the DEGs associated with hypertension might have a special role in stress-sensitive hypertension development in ISIAH rats.

The two most essential groups of DEGs related to regulation of biological processes were associated with regulation of blood pressure and blood circulation. Several other processes which may have an important role in hypertension development were: nitrogen compound biosynthetic process, ion transport, regulation of calcium ion transport, homeostatic process (including ion homeostasis and particularly calcium ion homeostasis), and tissue remodeling.

The functional annotation revealed multiple DEGs associated with the immune system process and with regulation of immune response. KEGG analysis also marked out several pathways significantly enriched with DEGs related to immune system function (Table 6). The other significantly enriched metabolic pathways were related to steroid hormone biosynthesis, tryptophan, glutathione, nitrogen, and drug metabolism. The DEGs in the identified KEGG pathways are listed in Additional file 3.

**Table 6:** Metabolic pathways significantly enriched with genes differentially expressed in ISIAH and WAG renal cortices

Term	Count	*P*-value
Complement and coagulation cascades	9	0.003
Type I diabetes mellitus	8	0.005
Cell adhesion molecules (CAMs)	13	0.006
Drug metabolism	8	0.014
Steroid hormone biosynthesis	6	0.018
Tryptophan metabolism	6	0.018
Metabolism of xenobiotics by cytochrome P450	7	0.020
Glutathione metabolism	6	0.032
Antigen processing and presentation	8	0.037
Nitrogen metabolism	4	0.047

## Discussion

It was well established that kidney is one of the target organs in hypertension development [13]. In the current study, RNA-Seq approach was performed to identify genes with altered transcriptional activity in the renal cortex of hypertensive ISIAH as compared to normotensive WAG rats and to reveal those, which might be responsible for hypertension development in ISIAH rats. Multiple DEGs associated with hypertension and renal diseases were found, and the functional annotation of DEGs helped to define the main biological processes and pathways which might contribute to stress-sensitive hypertension in ISIAH rats.

GO annotation results pointed out that the hormonal regulation might have strong influences on renal function. So, the group of 16 DEGs participating in the regulation of hormone level might play an important role in orchestration of the changes in physiological and metabolic processes in ISIAH kidney (Additional file 2). Several of them (*Ace, Comt, Cyp1a1, Glp1r, Hsd11b2*, and *Ren*) are widely known as associated with hypertension development.

The angiotensin I-converting enzyme and renin (*Ace* and *Ren*) are the key components of the renin-angiotensin system (RAS). Their low expression found in the current experiment is in a good agreement with the previous study when real-time PCR showed the decreased mRNA level of these genes in kidney cortex of 4-month old ISIAH males [14]. Low-renin hypertension usually implies increased retention of Na(+) [15]. As the statistically significant plasma sodium increase was found in ISIAH rats as compared to WAG [16], the low-renin hypertension in ISIAH rats may arise due to the suppression of the RAS by the sodium retention and elevated blood pressure.

*Comt* encodes the enzyme catechol-O-methyltransferase metabolizing catecholamines. The inhibition of COMT induces dopamine-dependent natriuresis [17]. The catechol-O-methyltransferase-gene-disrupted mice were resistant to salt-induced hypertension [18]. So, the decreased expression of *Comt* in the renal cortex of ISIAH rats may lead to increase in renal dopaminergic effects and sodium excretion, and may be considered as an adaptive mechanism. Earlier, the significantly decreased transcription of *Comt* was also detected in kidney of 6-month old ISIAH rats [19].

*Cyp1a1* knockout mice are hypertensive. Cyp1a1 metabolizes omega-3 polyunsaturated fatty acids to vasodilators and the loss of these vasodilators may lead to increases in BP [20]. So, the decreased level of *Cyp1a1* transcription in ISIAH renal cortex suggests its contribution to hypertension development in these rats.

The enzyme encoded by 11β-hydroxysteroid dehydrogenase (*Hsd11b2*) oxidizes glucocorticoids to the inactive metabolite cortisone. In aldosterone target tissues, 11βHSD2 is coexpressed with mineralocorticoid receptors and protects the receptor from activation by glucocorticoids. It was found that decreased HSD11B2 activity is related to hypertension [21] and *Hsd11b2* null mice are also hypertensive [22]. So, the increased *Hsd11b2* transcription in ISIAH renal cortex may lead to decreased glucocorticoid action and be protective against excessive elevation of blood pressure.

GLP-1 receptor (*Glp1r* gene) was shown to be expressed in glomerular capillary and vascular walls in the mouse kidney. Its signaling plays a crucial role in protection against increased renal oxidative stress [23]. So, *Glp1r* upregulation in renal cortex of ISIAH rats may be adaptive against the oxidative stress.

Several DEGs participating in the regulation of hormone level are related to retinol metabolism (*Cyp26b1, Rbp4,* and *Retsat*) and intracellular transport (*Rbp1*). Retinoids (vitamin A and its analogs) are highly potent regulators of cell differentiation, cell proliferation, and apoptosis. Retinoids and/or retinoid-related proteins play important role in the development of metabolic diseases, primarily obesity, diabetes, and dyslipidemia [24]. Earlier, several signs of metabolic syndrome, such as dislipidemia, increased glucose content, and increased body weight were described in ISIAH rats [25]. The elevated RBP4 was reported in chronic kidney disease [26] and may contribute to insulin resistance in spontaneously hypertensive rats [27]. Based on that, we suggest that the upregulation of *Rbp4* may be related to development of metabolic syndrome in ISIAH rats, too.

RetSat saturates all-trans-retinol to all-trans-13,14-dihydroretinol which is transiently oxidized to all-trans-13,14-dihydroretinoic acid before being oxidized further by Cyp26 enzymes [28]. Cyp26b1 catalyzes the inactivation of retinoic acid (RA) to hydroxylated forms and helps to maintain tissue RA concentrations within appropriate bounds [29]. The particular role of elevated transcription of *Retsat* and *Cyp26b1* in ISIAH kidney function remains to be determined.

According to the functional annotation, four genes responsible for regulation of hormone level *(Ace, Glp1r, Hsd11b2* and *Ren*) are also associated with the regulation of BP. Two other genes in the group ‘regulation of BP’ (*Ephx2*, *and Guca2b) were earlier described as* common genes related to regulation of BP in several rat models of programmed hypertension [30]. In ISIAH renal cortex these genes were upregulated. *Ephx2* encodes the soluble epoxide hydrolase (sEH) which metabolizes the epoxyeicosatrienoic acids (EETs) having antihypertensive properties. EETs also possess anti-inflammatory actions that could protect the kidney vasculature from injury during renal and cardiovascular diseases [31, 32]. sEH is considered as a main effector of angiotensin II-induced [31] and salt-sensitive hypertension [33]. Besides, it was considered as one of the gatekeeper genes contributing to programmed hypertension [30]. The uroguanylin (*Guca2b* encoded protein) deficiency results in impaired ability to excrete an enteral load of NaCl, primarily due to an inappropriate increase in renal Na + reabsorption, and in increased mean arterial pressure in uroguanylin knockout animals [34]. Based on this information, we may suggest that upregulation *of Ephx2* exerts the pressor effect and *Guca2b* exerts an opposite effect on systemic BP and renal function in ISIAH rats.

Three other DEGs related to regulation of BP (*Adra1b, P2rx4*, and *Ppara*) were downregulated in ISIAH renal cortex. The alpha1B-adrenoceptors (*Adra1b*) are involved in blood vessel remodeling [35] and mediate the vasoconstrictor actions of the renal sympathetic nerves in rats with renal failure [36, 37]. PPARα is a nuclear transcription factor. It contributes to regulation of BP and vascular reactivity in SHR [38]. PPARα deficiency appears to aggravate the severity of diabetic nephropathy through an increase in extracellular matrix formation, inflammation, and circulating free fatty acid and triglyceride concentrations [39]. Alternatively, the PPAR-alpha and -gamma agonists attenuate diabetic kidney disease in the apolipoprotein E knockout mice [40]. P2rx4(-/-) mice have higher BP and excrete smaller amounts of NO products in their urine than do wild-type mice. They have impaired flow-dependent control of vascular tone and remodeling [41]. Besides, the lack of P2X4R expression leads to increased renal fibrosis [42]. So, we may suggest that downregulation of *Adra1b* may be adaptive and protect against the excessive sympathoexcitation, and *P2rx4*, and *Ppara* deficiency may contribute to development of kidney pathology in ISIAH rats.

In the current study, we found multiple DEGs related to response to different stimuli and to stress. This is well consistent with the previous observation that HPA and sympathoadrenal systems are activated in ISIAH rats [11] and that the changes in kidney function of 6-month old ISIAH rats are based on altered expression of many genes working in stress-related mode [19]. The stress response (or stress cascade) is considered as disruptions in homeostasis which result in a series of neural and endocrine adaptations. The stress cascade is responsible for allowing the body to make the necessary physiological and metabolic changes required to cope with the demands of a homeostatic challenge [43]. In the current study, the functional annotation helped to identify multiple DEGs involved in homeostatic process. Several DEGs in this group were associated with both hypertension and kidney diseases. That is in a good agreement with the opinion that essential hypertension is one of the “syndromes of impaired genetic homeostasis” [44] and that homeostatic process might be impaired in patients with chronic kidney disease [45].

Earlier, the comparative electron microscopic study of glomerular apparatus in 6-month old ISIAH and Wistar rats showed hypertrophy of renal corpuscles in hypertensive kidney, accompanied by multiple structural changes such as capillary narrowing or dilation, endothelial flattening, podocyte hypertrophy and flattening of their cytopodia, thickening of basal lamina, mesangial volume expansion and increase in the number of intercapillary processes of mesangial cells [9]. Complex of these signs suggested a disturbance of glomerular capillary blood circulation and a functional podocyte stress, compensating the microcirculatory disturbances. Changes in basal membranes and mesangium were considered as indicative of increase in filtration barrier functional load, and of initial stages of glomerular sclerosis [9]. In the current study, we used younger rats, nevertheless, we found groups of DEGs associated with blood circulation, renal hypertension, and with the development of nephrosclerosis. Probably, the changes in their expression may be potentially important for the appearance of the microcirculatory and structural disturbances in aging kidney.

The particular types of stress specified by GO terms were associated with oxidative stress, hypoxia, and fluid shear stress (Additional file 1). The oxidative stress is considered to be the pathogenic outcome of oxidant overproduction, which occurs as a result of imbalance between prooxidants and antioxidants [46]. Several genes in this group showed reduced transcription and several were upregulated.

The protein encoded by *Abcc2* mediates transport of various molecules across extra- and intra-cellular membranes, including the transport of prostaglandin E2 [47], which affects multiple segments of the preglomerular vascular tree in a different ways [48]. ABCC2 deficiency may be associated with increased oxidative stress, leading to renal tubular cell damage [49].

Two other genes associated with response to oxidative stress (*Clu* and *Mmp9*) are known as genes related to hypertension and kidney diseases. Clusterin (*Clu*) upregulation attenuates renal fibrosis in obstructive nephropathy [50]. Alternatively, the loss of clusterin expression worsens renal ischemia-reperfusion injury [51]. Over-expression of MMP9 could alter glomerular basement membrane components thereby causing podocyte structural changes [52]. MMP9 is also known to cleave podocalyxin in podocytes, which is a charge barrier to prevent microalbuminuria [53]. Loss of MMP9 reduces atherosclerotic burden [54] and, alternatively, the elevated urine values of MMP-9 was recognized as a marker of atherosclerotic disease [55]. So, the decrease in *Clu* and *Mmp9* expression may be protective in ISIAH renal cortex.

Another gene repressed in ISIAH renal cortex and related to oxidative stress was *Hao1.* It encodes a peroxisomal enzyme that oxidizes glycolate to glyoxylate with concomitant production of H_2_O_2_. Downregulation of Hao1 expression during oxidative stress may provide a mechanism to prevent excessive H_2_O_2_ formation [56]. Alpha B-crystallin (*Cryab* gene) is a ubiquitous stress inducible molecular chaperone. CRYAB is promoting angiogenesis and preventing apoptosis [57]. Expression of cystatin C has protective effects against various oxidative stresses that induce cell death [58]. Its decreased transcription in ISIAH renal cortex may contribute to oxidative damage and, probably, to hypertension development.

It is known that chronic hypertension can occur if there is an abnormality of kidney function that shifts pressure natriuresis so that sodium balance is maintained at elevated blood pressures [59]. Tubular sodium reabsorption depends on the activity of ion transport systems, which are modulated by neural, endocrine, paracrine, and physical factors [60]. In our study, the functional annotation revealed 32 DEGs related to ion transport. The changes in their transcriptional activity suggest that different mechanisms of osmoregulation may contribute to function of hypertensive kidney. These results are in a good agreement with the statement that ion transport is one of the major processes that are vital for functions of kidney and organism as a whole.

KEGG analysis showed an overrepresentation of DEGs involved in several metabolic pathways (Table 6 and Additional file 3). The most significantly enriched one was pathway associated with complement and coagulation cascades. Inflammation and coagulation play pivotal roles in the pathogenesis of vascular diseases. Increasing evidence points to an extensive cross-talk between these two systems, whereby inflammation leads not only to activation of coagulation, but coagulation also considerably affects inflammatory activity [61].

One of the DEGs related to this pathway (*Fga*) is involved in platelet aggregation and has been recognized as biomarker for acute kidney injury [62]. Its upregulation may contribute to enhanced coagulation and exert negative effect on ISIAH kidney function. The other upregulated gene related to complement and coagulation cascades (*Serpinc1*) contributes to negative regulation of inflammatory response and to fibrinolysis. *Serpinc1* deficiency is significantly associated with a tendency toward thrombosis formation in the kidney [63]. So, its upregulation may exert a protective effect on ISIAH kidney function.

Two another upregulated DEGs (*Klkb1* and *Vwf*) in the complement and coagulation cascades are known as associated with both hypertension and kidney diseases. Plasma prekallikrein (*Klkb1* gene) was considered as a risk marker for hypertension and nephropathy in type 1 diabetes. Its level was elevated in association with increased blood pressure, and positively correlated with urinary albumin excretion rate [64]. As for *Vwf* gene, it was demonstrated that immobilization stress exposure was followed by a rise in von Willebrand factor concentrations, adrenocorticotropic hormone and corticosterone release in saline pretreated rats [65]. The enhanced *Vwf* gene transcription in ISIAH renal cortex suggests that it may be one of the genes working in stress-related mode in renal cortex of ISIAH rats.

Several pathways found in the current study were closely related to the immune system function. It is long known that the immune system changes play a role in hypertension and an extensive bidirectional interactions between the sympathetic nervous system and the immune system exist [66, 67]. Recent studies have shown that both innate and adaptive immunity contribute to hypertension [68]. Major histocompatibility complex (MHC) class I molecules are ligands for the killer-cell immunoglobulin-like receptors, which are expressed by natural killer (NK) cells and T cells. The interactions between these molecules contribute to both innate and adaptive immunity [69]. MHC class-II molecules are key participants in immune activation events in autoimmunity [70]. It was shown that mice lacking adaptive immune cells, including recombinase-activating gene-deficient mice and rats and mice with severe combined immunodeficiency have blunted hypertension to stimuli such as ANG II, high salt, and norepinephrine [71]. The current work helped to reveal several genes related to MHC class I and MHC class II which might be useful for further studies of immune system changes during hypertension development in ISIAH rats.

Several other pathways were significantly enriched with DEGs related to steroid hormone biosynthesis, tryptophan, glutathione, nitrogen, and drug metabolism. The most DEGs associated with hypertension in these pathways were related to steroid hormone biosynthesis and were discussed above. The other pathways (glutathione, nitrogen, and drug metabolism) didn’t contain the DEGs directly associated with hypertension, however, these also might play important role in pathology development in ISIAH rats. This may be true, at least for the glutathione metabolism. It is known that glutathione is an important intracellular antioxidant that protects against a variety of different oxidant species. Induction of oxidative stress by glutathione depletion causes severe hypertension in normal rats [72]. In our study, we found several DEGs involved in glutathione metabolism. GPx2 is a key enzyme in the antioxidant system of the cells [73]. The glutathione S-transferases provide cellular protection against the toxic effects of a number of environmental toxicants and products of oxidative stress by conjugation with glutathione [74]. So, we may suggest that downregulation of glutathione S-transferases may weaken oxidative defense and mediate the pathological processes in ISIAH kidney and the upregulation of *Gpx2* seems to play a protective role.

## Conclusion

The results of the study revealed multiple genes differentially expressed in renal cortex of hypertensive ISIAH and normotensive WAG rats, including 42 genes associated with hypertension and regulation of BP. Their functional annotation showed that many different processes might be brought into play. Two DEGs associated with hypertension (*Ephx2 and Glp1r*) were in the list of the top 40 genes showing the highest differences in expression in ISIAH and WAG renal cortex. These DEGs may be considered as potential candidates for further studies to better understand the mechanisms of the hypertension development in the ISIAH rats. The results of the discussion suggested that the interstrain differences in ISIAH and WAG renal function may probably arise from the imbalance in processes leading to the development of pathology from one side and the processes trying to restore the homeostasis from the other side. As the number of hypertensive and the other potentially relevant genes was considerable, we were not able to discuss all of them in details.

Our findings provide a basis for identification of potential biomarkers of stress-sensitive hypertension and further investigation of the signaling mechanisms that affect kidney function and contribute to hypertension development.

## Methods

### Animals

The work was carried out on hypertensive ISIAH and normotensive WAG rats bred in the Center for Genetic Resources of Laboratory Animals at the Institute of Cytology and Genetics, Siberian Branch of the Russian Academy of Sciences, (Novosibirsk, Russia, RFMEFI61914X0005 and RFMEFI61914X0010). All rats were maintained in the standard conditions with free access to food and water. The systolic arterial blood pressure (BP) was measured indirectly by the tail-cuff method. The BP level was determined under short-term ether anesthesia to exclude the effect of psychological stress induced by the measuring procedure. In RNA-seq experiments the 3-month old ISIAH (*n* = 3), and WAG (*n* = 3) males were used. Their systolic arterial BP was 171.7. ± .1.22 mmHg in ISIAH and 116.33 ± 1.86 mmHg in WAG males. The kidney of the decapitated rats was immediately removed and sectioned to get the samples of renal cortex which were stored in RNA Later (Qiagen, Chatsworth, CA) at −70 °C until use. All animal experiments were approved by the Institute’s Animal Care and Use Committee.

### RNA-seq analysis

The collected samples of renal cortex were sent to JSC Genoanalytica (Moscow, Russia), where mRNA was extracted using Dynabeads mRNA Purification Kit (Ambion, USA). cDNA libraries were constructed using NEBNext mRNA Library Prep Reagent Set for Illumina (NEB, USA) following the manufacturer’s protocol and were subjected to Illumina single-end sequencing. Three renal cortex samples from ISIAH and three renal cortex samples from WAG rats were run as experimental replicates. The resulting fastq-formatted sequencing data were mapped to the RGSC Rnor_5.0\rn5 reference genome using Tophat2 aligner [75] and NCBI RefSeq gene annotation. Quality assessment of the mapped data was performed using the module ‘CollectRnaSeqMetrics’ from Picard tools suite (http://broadinstitute.github.io/picard/). The summary statistics for each sequenced library is given in Additional file 4. The Cufflinks/Cuffdiff programs were then used to estimate gene expression levels in FPKM (fragments per kilobase of transcript per million mapped reads), and to perform differential expression analysis [76]. Genes were considered to be differentially expressed at q value < 0,05.

### Functional annotation

The functional analysis of DEGs was performed using DAVID (The Database for Annotation, Visualization and Integrated Discovery) tool (http://david.abcc.ncifcrf.gov/) [77, 78]. The Gene Ontology option was utilized to determine the significantly (*p* < 0.05) enriched biological processes and groups of genes possibly contributing to hypertensive phenotype in ISIAH rats. The Kyoto Encyclopedia of Genes and Genomes Pathway Database (KEGG, http://www.genome.jp/kegg/) was used to identify pathways that were most significant to the data set. The genes related to hypertension and renal diseases were detected according to the DEGs annotation in Rat Genome Database (RGD, http://rgd.mcw.edu/). The detection of transcription factors among DEGs was performed using gene annotations from GenBank (http://www.ncbi.nlm.nih.gov/gene/), an atlas of combinatorial transcriptional regulation in mouse and man [79] and Panther classification system (http://www.pantherdb.org/).

### Quantitative real-time PCR (qPCR)

The relative amount of target mRNA was measured by qPCR. Samples of renal cotrtex were analyzed in 3-month old ISIAH and WAG rats. Each group contained five rats. Total RNA was extracted using the TRI reagent (Molecular research center, USA). Remaining traces of genomic DNA were removed from the RNA samples using DNase I (Promega, USA) treatment, according to the manufacturer’s instructions.

Reverse transcription was performed in 50 μl of RT buffer containing 3 μg of total RNA, 0.25 nmol of random nonanucleotide primers (Biosan, Russia), 0.4 mM dNTP, and 40 units of MoMLV (Vektor-Best, Russia). The cDNA was synthesized at 37 °C (1 h), 42 °C (30 min), and 50 °C (10 min). The enzyme was inactivated by heating the mixture at 75 °C for 5 min.

qPCR was performed in a final volume of 20 μl. The reaction volume contained master mix with SYBR Green, forward and reverse primers (0,15 mM each), 1 unit of HotStart Taq polymerase (Vektor-Best, Russia), and the cDNA template. The housekeeping gene *Rpl30* encoding ribosomal protein L30 was used as a reference gene. Primer’s sequences, their annealing temperatures, and the temperatures of fluorescence signal acquisition are given in Additional file 5.

qPCR was carried out in an iCycler iQ4 Real-Time PCR Detection System (Bio-Rad Laboratories, USA) with an initial denaturation of 1 min at 94 °C followed by 40 cycles of 15 s at 94 °C, 20 s at primer’s annealing temperatures (see Additional file 5), 20 s at 72 °C, fluorescence signal acquisition for 10 s, and then generation of melting curve from 65 to 94 °C. The standard-curve quantitation method was applied [80]: the relative amount of the tested cDNA was determined using calibration curves derived from the dilutions of the standard cDNA. Standard cDNA solution for plotting calibration curves was obtained by mixing aliquots from each of the synthesized cDNA samples. In each experiment, cDNA samples with primers for the target gene (four replicates per cDNA sample), the same samples with primers for the reference gene (four replicates), and the standard cDNA dilutions (1 : 1, 1 : 4, 1 : 16, and 1 : 64) with the primers for the target gene (two replicates), and with the primers for the reference gene (two replicates) were placed on the same plate. The value for the target gene was further normalized against the qPCR level of the reference gene.

Statistical calculations for qPCR data were performed with the software package Statistica v.6.0 (Statsoft, USA) using nonparametric statistics, Mann-Whitney U-test. Differences were considered statistically significant when P was less than 0.05. The data were presented as means and their standard errors (M ± S.E.M.).

## Availability of supporting data

The data sets supporting the results of this article are included within the article and its additional files.

## Additional files

Additional file 1:
**GO Terms and DEGs.** (XLS 188 kb) Additional file 2:
**DEGs related to GO terms.** (XLS 92 kb) Additional file 3:
**DEGs in the KEGG pathways.** (XLS 27 kb) Additional file 4:
**The number of mapped reads in hypothalamuses of ISIAH and WAG rats*.** (XLS 22 kb) Additional file 5:
**Primers used in real-time PCR.** (DOC 36 kb)

## Abbreviations

AA: Arachidonic acid
BP: Blood pressure
DAVID: Database for Annotation, Visualization and Integrated Discovery
DEG: Differentially expressed genes
EETs: Epoxyeicosatrienoic acids
FPKM: Fragments per kilobase of transcript per million mapped reads
GO: Gene Ontology
HPA: Hypothalamic-pituitary-adrenal
ISIAH: Inherited Stress-Induced Arterial Hypertension
KEGG: Kyoto Encyclopedia of Genes and Genomes Pathway Database
MHC: Major histocompatibility complex
NK: Natural killer
qPCR: Quantitative real time polymerase chain reaction
RA: retinoic acid
RAS: renin-angiotensin system
RGD: Rat Genome Database
RNA-seq: RNA sequencing
SABP: Systolic arterial blood pressure
sEH: Soluble epoxide hydrolase
WAG: Wistar Albino Glaxo

## References

[1] Hall JE (2003). The kidney, hypertension, and obesity. Hypertension.

[2] Mullins LJ, Bailey MA, Mullins JJ (2006). Hypertension, kidney, and transgenics: a fresh perspective. Physiol Rev.

[3] Turak O, Ozcan F, Tok D, Isleyen A, Sokmen E, Tasoglu I (2013). Serum uric acid, inflammation, and nondipping circadian pattern in essential hypertension. J Clin Hypertens (Greenwich).

[4] Dornas WC, Silva ME (2011). Animal models for the study of arterial hypertension. J Biosci.

[5] Adarichev VA, Korokhov NP, Ostapchuk Ia V, Dymshits GM, Markel AL (1996). Characterization of rat lines with normotensive and hypertensive status using genomic fingerprinting. Genetika.

[6] Markel AL, Sassard J (1992). Development of a new strain of rats with inherited stress-induced arterial hypertension. Genetic hypertension.

[7] Markel AL, Maslova LN, Shishkina GT, Bulygina VV, Machanova NA, Jacobson GS, McCarty R, Blizard DA, Chevalier RL (1999). Developmental influences on blood pressure regulation in ISIAH rats. Development of the hypertensive phenotype: basic and clinical studies.

[8] Redina OE, Machanova NA, Efimov VM, Markel AL (2006). Rats with inherited stress-induced arterial hypertension (ISIAH strain) display specific quantitative trait loci for blood pressure and for body and kidney weight on chromosome 1. Clin Exp Pharmacol Physiol.

[9] Shmerling MD, Filiushina EE, Lazarev VA, Buzueva II, Markel’ AL, Iakobson GS (2001). Ultrastructural changes of kidney corpuscles in rats with hereditary stress-induced arterial hypertension [Article in Russian]. Morfologiia.

[10] Filyushina EE, Shmerling MD, Buzueva II, Lazarev VA, Markel AL, Yakobson GS (2013). Structural characteristics of renomedullary interstitial cells of hypertensive ISIAH rats. Bull Exp Biol Med.

[11] Markel AL, Redina OE, Gilinsky MA, Dymshits GM, Kalashnikova EV, Khvorostova YV (2007). Neuroendocrine profiling in inherited stress-induced arterial hypertension rat strain with stress-sensitive arterial hypertension. J Endocrinol.

[12] Marguerat S, Bahler J (2010). RNA-seq: from technology to biology. Cell Mol Life Sci.

[13] Mensah GA, Croft JB, Giles WH (2002). The heart, kidney, and brain as target organs in hypertension. Cardiol Clin.

[14] Fedoseeva LA, Antonov EV, Klimov LO, Dymshits GM, Markel AL, Himura A, Sato T (2013). Function of the renin-angiotensin-aldosterone system in the ISIAH rats with stress-sensitive arterial hypertension. Renin-angiotensin system: physiology, role in disease and health implications.

[15] Pratt JH (2000). Low-renin hypertension: more common than we think?. Cardiol Rev.

[16] Fedoseeva LA, Riazanova MA, Antonov EV, Dymshits GM, Markel’ AL (2011). Renin-angiotensin system gene expression in the kidney and in the heart in hypertensive ISIAH rats. [Article in Russian]. Biomed Khim.

[17] Eklof AC, Holtback U, Sundelof M, Chen S, Aperia A (1997). Inhibition of COMT induces dopamine-dependent natriuresis and inhibition of proximal tubular Na+, K + -ATPase. Kidney Int.

[18] Helkamaa T, Mannisto PT, Rauhala P, Cheng ZJ, Finckenberg P, Huotari M (2003). Resistance to salt-induced hypertension in catechol-O-methyltransferase-gene-disrupted mice. J Hypertens.

[19] Redina OE, Smolenskaya SE, Abramova TO, Ivanova LN, Markel AL (2015). Differential transcriptional activity of kidney genes in hypertensive ISIAH and normotensive WAG rats. Clin Exp Hypertens.

[20] Agbor LN, Walsh MT, Boberg JR, Walker MK (2012). Elevated blood pressure in cytochrome P4501A1 knockout mice is associated with reduced vasodilation to omega-3 polyunsaturated fatty acids. Toxicol Appl Pharmacol.

[21] Friso S, Pizzolo F, Choi SW, Guarini P, Castagna A, Ravagnani V (2008). Epigenetic control of 11 beta-hydroxysteroid dehydrogenase 2 gene promoter is related to human hypertension. Atherosclerosis.

[22] Evans LC, Livingstone DE, Kenyon CJ, Jansen MA, Dear JW, Mullins JJ (2012). A urine-concentrating defect in 11?-hydroxysteroid dehydrogenase type 2 null mice. Am J Physiol Renal Physiol.

[23] Fujita H, Morii T, Fujishima H, Sato T, Shimizu T, Hosoba M (2014). The protective roles of GLP-1R signaling in diabetic nephropathy: possible mechanism and therapeutic potential. Kidney Int.

[24] Brun PJ, Yang KJ, Lee SA, Yuen JJ, Blaner WS (2013). Retinoids: potent regulators of metabolism. Biofactors.

[25] Pivovarova EN, Dushkin MI, Perepechaeva ML, Kobzev VF, Trufakin VA, Markel’ AL (2011). All signs of metabolic syndrome in the hypertensive ISIAH rats are associated with increased activity of transcription factors PPAR, LXR, PXR, and CAR in the liver. [Article in Russian]. Biomed Khim.

[26] Frey SK, Nagl B, Henze A, Raila J, Schlosser B, Berg T (2008). Isoforms of retinol binding protein 4 (RBP4) are increased in chronic diseases of the kidney but not of the liver. Lipids Health Dis.

[27] Ou HY, Wu HT, Yang YC, Wu JS, Cheng JT, Chang CJ (2011). Elevated retinol binding protein 4 contributes to insulin resistance in spontaneously hypertensive rats. Horm Metab Res.

[28] Moise AR, Isken A, Dominguez M, de Lera AR, von Lintig J, Palczewski K (2007). Specificity of zebrafish retinol saturase: formation of all-trans-13,14-dihydroretinol and all-trans-7,8- dihydroretinol. Biochemistry.

[29] Ross AC, Zolfaghari R (2011). Cytochrome P450s in the regulation of cellular retinoic acid metabolism. Annu Rev Nutr.

[30] Tain YL, Huang LT, Chan JY, Lee CT (2015). Transcriptome analysis in rat kidneys: importance of genes involved in programmed hypertension. Int J Mol Sci.

[31] Jung O, Brandes RP, Kim IH, Schweda F, Schmidt R, Hammock BD (2005). Soluble epoxide hydrolase is a main effector of angiotensin II-induced hypertension. Hypertension.

[32] Imig JD (2005). Epoxide hydrolase and epoxygenase metabolites as therapeutic targets for renal diseases. Am J Physiol Renal Physiol.

[33] Imig JD, Zhao X, Zaharis CZ, Olearczyk JJ, Pollock DM, Newman JW (2005). An orally active epoxide hydrolase inhibitor lowers blood pressure and provides renal protection in salt-sensitive hypertension. Hypertension.

[34] Lorenz JN, Nieman M, Sabo J, Sanford LP, Hawkins JA, Elitsur N (2003). Uroguanylin knockout mice have increased blood pressure and impaired natriuretic response to enteral NaCl load. J Clin Invest.

[35] Zhang H, Cotecchia S, Thomas SA, Tanoue A, Tsujimoto G, Faber JE (2004). Gene deletion of dopamine beta-hydroxylase and alpha1-adrenoceptors demonstrates involvement of catecholamines in vascular remodeling. Am J Physiol Heart Circ Physiol.

[36] Khan AH, Sattar MA, Abdullah NA, Johns EJ (2007). Influence of cisplatin-induced renal failure on the alpha(1)-adrenoceptor subtype causing vasoconstriction in the kidney of the rat. Eur J Pharmacol.

[37] Hye Khan MA, Sattar MA, Abdullah NA, Johns EJ (2008). Influence of combined hypertension and renal failure on functional alpha(1)-adrenoceptor subtypes in the rat kidney. Br J Pharmacol.

[38] Yousefipour Z, Newaz M (2014). PPARalpha ligand clofibrate ameliorates blood pressure and vascular reactivity in spontaneously hypertensive rats. Acta Pharmacol Sin.

[39] Park CW, Kim HW, Ko SH, Chung HW, Lim SW, Yang CW (2006). Accelerated diabetic nephropathy in mice lacking the peroxisome proliferator-activated receptor alpha. Diabetes.

[40] Calkin AC, Giunti S, Jandeleit-Dahm KA, Allen TJ, Cooper ME, Thomas MC (2006). PPAR-alpha and -gamma agonists attenuate diabetic kidney disease in the apolipoprotein E knockout mouse. Nephrol Dial Transplant.

[41] Yamamoto K, Sokabe T, Matsumoto T, Yoshimura K, Shibata M, Ohura N (2006). Impaired flow-dependent control of vascular tone and remodeling in P2X4-deficient mice. Nat Med.

[42] Kim MJ, Turner CM, Hewitt R, Smith J, Bhangal G, Pusey CD (2014). Exaggerated renal fibrosis in P2X4 receptor-deficient mice following unilateral ureteric obstruction. Nephrol Dial Transplant.

[43] Miller DB, O’Callaghan JP (2002). Neuroendocrine aspects of the response to stress. Metabolism.

[44] Neel JV, Weder AB, Julius S (1998). Type II diabetes, essential hypertension, and obesity as “syndromes of impaired genetic homeostasis”: the “thrifty genotype” hypothesis enters the 21st century. Perspect Biol Med.

[45] White WB (2007). Cardiovascular effects of the cyclooxygenase inhibitors. Hypertension.

[46] Touyz RM (2004). Reactive oxygen species, vascular oxidative stress, and redox signaling in hypertension: what is the clinical significance?. Hypertension.

[47] de Waart DR, Paulusma CC, Kunne C, Oude Elferink RP (2006). Multidrug resistance associated protein 2 mediates transport of prostaglandin E2. Liver Int.

[48] van Rodijnen WF, Korstjens IJ, Legerstee N, Ter Wee PM, Tangelder GJ (2007). Direct vasoconstrictor effect of prostaglandin E2 on renal interlobular arteries: role of the EP3 receptor. Am J Physiol Renal Physiol.

[49] Grisk O, Steinbach AC, Ciecholewski S, Schluter T, Kloting I, Schmidt H (2009). Multidrug resistance-related protein 2 genotype of the donor affects kidney graft function. Pharmacogenet Genomics.

[50] Jung GS, Kim MK, Jung YA, Kim HS, Park IS, Min BH (2012). Clusterin attenuates the development of renal fibrosis. J Am Soc Nephrol.

[51] Zhou W, Guan Q, Kwan CC, Chen H, Gleave ME, Nguan CY (2010). Loss of clusterin expression worsens renal ischemia-reperfusion injury. Am J Physiol Renal Physiol.

[52] Lelongt B, Legallicier B, Piedagnel R, Ronco PM (2001). Do matrix metalloproteinases MMP-2 and MMP-9 (gelatinases) play a role in renal development, physiology and glomerular diseases?. Curr Opin Nephrol Hypertens.

[53] Fernandez D, Larrucea S, Nowakowski A, Pericacho M, Parrilla R, Ayuso MS (1813). Release of podocalyxin into the extracellular space. Role of metalloproteinases. Biochim Biophys Acta.

[54] Luttun A, Lutgens E, Manderveld A, Maris K, Collen D, Carmeliet P (2004). Loss of matrix metalloproteinase-9 or matrix metalloproteinase-12 protects apolipoprotein E-deficient mice against atherosclerotic media destruction but differentially affects plaque growth. Circulation.

[55] Fitzsimmons PJ, Forough R, Lawrence ME, Gantt DS, Rajab MH, Kim H (2007). Urinary levels of matrix metalloproteinase 9 and 2 and tissue inhibitor of matrix metalloproteinase in patients with coronary artery disease. Atherosclerosis.

[56] Recalcati S, Tacchini L, Alberghini A, Conte D, Cairo G (2003). Oxidative stress-mediated down-regulation of rat hydroxyacid oxidase 1, a liver-specific peroxisomal enzyme. Hepatology.

[57] Campbell-Lloyd AJ, Mundy J, Deva R, Lampe G, Hawley C, Boyle G (2013). Is alpha-B crystallin an independent marker for prognosis in lung cancer?. Heart Lung Circ.

[58] Nishiyama K, Konishi A, Nishio C, Araki-Yoshida K, Hatanaka H, Kojima M (2005). Expression of cystatin C prevents oxidative stress-induced death in PC12 cells. Brain Res Bull.

[59] Hall JE, Louis K (1994). Dahl Memorial Lecture. Renal and cardiovascular mechanisms of hypertension in obesity. Hypertension.

[60] Strazzullo P, Galletti F, Barba G (2003). Altered renal handling of sodium in human hypertension: short review of the evidence. Hypertension.

[61] Levi M, van der Poll T, Buller HR (2004). Bidirectional relation between inflammation and coagulation. Circulation.

[62] Hoffmann D, Bijol V, Krishnamoorthy A, Gonzalez VR, Frendl G, Zhang Q (2012). Fibrinogen excretion in the urine and immunoreactivity in the kidney serves as a translational biomarker for acute kidney injury. Am J Pathol.

[63] Yanada M, Kojima T, Ishiguro K, Nakayama Y, Yamamoto K, Matsushita T (2002). Impact of antithrombin deficiency in thrombogenesis: lipopolysaccharide and stress-induced thrombus formation in heterozygous antithrombin-deficient mice. Blood.

[64] Jaffa AA, Durazo-Arvizu R, Zheng D, Lackland DT, Srikanth S, Garvey WT (2003). Plasma prekallikrein: a risk marker for hypertension and nephropathy in type 1 diabetes. Diabetes.

[65] Jezova D, Kristova V, Slamova J, Mlynarik M, Pirnik Z, Kiss A (2003). Stress-induced rise in endothelaemia, von Willebrand factor and hypothalamic-pituitary-adrenocortical axis activation is reduced by pretreatment with pentoxifylline. J Physiol Pharmacol.

[66] Purcell ES, Gattone VH (1992). Immune system of the spontaneously hypertensive rat. I. Sympathetic innervation. Exp Neurol.

[67] Fu ML (1995). Do immune system changes have a role in hypertension?. J Hypertens.

[68] Harrison DG, Vinh A, Lob H, Madhur MS (2010). Role of the adaptive immune system in hypertension. Curr Opin Pharmacol.

[69] Parham P (2005). MHC class I molecules and KIRs in human history, health and survival. Nat Rev Immunol.

[70] Nepom GT, Erlich H (1991). MHC class-II molecules and autoimmunity. Annu Rev Immunol.

[71] Trott DW, Harrison DG (2014). The immune system in hypertension. Adv Physiol Educ.

[72] Vaziri ND, Wang XQ, Oveisi F, Rad B (2000). Induction of oxidative stress by glutathione depletion causes severe hypertension in normal rats. Hypertension.

[73] Pigeolet E, Remacle J (1991). Susceptibility of glutathione peroxidase to proteolysis after oxidative alteration by peroxides and hydroxyl radicals. Free Radic Biol Med.

[74] Hayes JD, Flanagan JU, Jowsey IR (2005). Glutathione transferases. Annu Rev Pharmacol Toxicol.

[75] Kim D, Pertea G, Trapnell C, Pimentel H, Kelley R, Salzberg SL (2013). TopHat2: accurate alignment of transcriptomes in the presence of insertions, deletions and gene fusions. Genome Biol.

[76] Trapnell C, Hendrickson DG, Sauvageau M, Goff L, Rinn JL, Pachter L (2013). Differential analysis of gene regulation at transcript resolution with RNA-seq. Nat Biotechnol.

[77] Huang DW, Sherman BT, Lempicki RA (2009). Systematic and integrative analysis of large gene lists using DAVID bioinformatics resources. Nat Protoc.

[78] Huang DW, Sherman BT, Lempicki RA (2009). Bioinformatics enrichment tools: paths toward the comprehensive functional analysis of large gene lists. Nucleic Acids Res.

[79] Ravasi T, Suzuki H, Cannistraci CV, Katayama S, Bajic VB, Tan K (2010). An atlas of combinatorial transcriptional regulation in mouse and man. Cell.

[80] Ginzinger DG (2002). Gene quantification using real-time quantitative PCR: an emerging technology hits the mainstream. Exp Hematol.

