# Supplementary material for: Comparative transcriptional profiling of renal cortex in rats with inherited stress-induced arterial hypertension and normotensive Wistar Albino Glaxo rats
Source: BMC Genet. 2016 Jan 27;17(Suppl 1):12. doi: 10.1186/s12863-015-0306-9 (PMC4895253; doi:10.1186/s12863-015-0306-9)
Supplement: Additional file 5: — Primers used in real-time PCR. (DOC 36 kb) [file 12863_2015_306_MOESM5_ESM.doc]

**Additional file 5.**

Primers used in real-time PCR.

| Gene | Primers, 5′→3′ | T_anneal_,  ^o^C | T_acq_,  ^o^C | Length of  PCR fragment, bp |
| --- | --- | --- | --- | --- |
| *Comt* | F: CTTGACCACTGGAAAGACCG  R: CGATGACGTTGTCAGCTAGGA | 61 | 84 | 100 |
| *Ephx2* | F: TTTCTTGGAGGTACCAG↓ATCC  R: CAGTCATGGCCAATGAACAC | 62 | 84 | 193 |
| *Ppara* | F: AATTTGCTGTGGAG↓ATCGGC  R: TGGGAAGAGAAAGGTATCATC | 64 | 85 | 127 |
| *Hpgd* | F: TATTTCTTCAATAGCAG↓GGCTCA  R: TCAGTCTCACACCGCTTTTC | 63 | 84 | 131 |
| *Adra1b* | F: CCAAAACCTTGGGCATTGTA  R: TAGATGATGGGATTGAGGCA | 64 | 87 | 166 |
| *Rpl30* | F: ATGGTGGCTGCAAAGAAGAC  R: CAAAGCTGGACAGTTGTTGG | 61-65* | 84 | 143 |

↓ position of exon–exon junction site in mRNA; *T*_anneal_ – the annealing temperature used in experiment, *T*_acq_ - the temperature of fluorescence signal acquisition. *– the range of appropriate annealing temperatures for reference gene. F – forward primer; R - reverse primer.
